# Zeolite-based monoliths for water softening by ion exchange/precipitation process

**DOI:** 10.1038/s41598-022-07679-2

**Published:** 2022-03-07

**Authors:** A. Campanile, B. Liguori, C. Ferone, D. Caputo, P. Aprea

**Affiliations:** 1 Applied Chemistry Labs-Department of Chemical, Materials and Industrial Engineering, University of Naples Federico II, Naples, Italy; 2 Materials Science and Engineering Research Group MASERG, Department of Engineering, University of Naples Parthenope, Naples, Italy

**Keywords:** Materials science, Pollution remediation

## Abstract

In this work, the design of a monolithic softener obtained by geopolymer gel conversion is proposed. The softener used consists in a geopolymeric macroporous matrix functionalized by the co-crystallization of zeolite A and X in mixture. The dual nature of the proposed material promotes a softening process based on the synergistic effect of cation exchange and alkaline precipitation. A softening capacity of 90% and 54% for Ca^2+^ and Mg^2+^ respectively was attained in 24 h. In fact, the softener reported a Cation Exchange Capacity (CEC) value of 4.43 meq g^−1^. Technical features such as density, porosity and mechanical resistance were also measured. The use of this monolithic softener can improve performance and sustainability of hardness removal from tap water, reducing the production of sludge and adding the possibility to partially regenerate or reuse it.

## Introduction

The total concentration of magnesium and calcium in water, known as water hardness, represents a great problem for all the industrial processes which use water for steam generation, such as textile, paper, dying industry. Direct feed of hard water to the boiler reduces the steam production due to the presence of metal ions, which, forming scale and sludge, priming and foaming, can greatly reduce the heat transfer efficiency^1^. Moreover, the scale formation during industrial activities produces boiler corrosion and blokage of membranes and pipes^2^. Hence the necessity to soften the water prior to use in such applications. Water softening can be mainly performed by means of several treatments^3–5^, such as electrolysis^6^, microbial and electrochemical processes^7,8^, nanofiltration^7,9^, adsorption^10,11^, chemical precipitation^12–14^ and ion exchange^15^. Among the chemical precipitation methods, lime softening, involving the use of specific compounds that help in precipitation of Ca^2+^ and Mg^2+^ ions by removing water hardness, is still known as a cost-effective treatment in some applications^16^. Frequently, the use of a combination of both lime softening and cationic ion exchange units allows to achieve minimum hardness^16^. Other methods based on ion exchange resins have become more interesting, being continuous processes that also allow regeneration operations. Zeolites, thanks to their excellent ion-exchange and sorption properties^17^, are employed to a large extent as water softeners^18,19^. In particular, zeolites Na-A and Na-X can exchange their sodium ions with “hard water ions”, such as calcium and magnesium^20^. It has been also demonstrated that a synergistic effect can be reached using a mixture of them^21^. In order to overcome the main drawback of this batch-wise use of zeolites, that is the disposal of saturated adsorbents, a zeolitic membrane can be used. Nowadays, zeolite membranes are principally obtained by grow crystal seeds previously deposited on a proper substrate. The technological problem linked to this method is to obtain an uniform distribution of the crystallization seeds on the substrate which therefore causes some defects^22^. Geopolymer Gel Conversion (GGC) represents an innovative and sustainable method to structuring powdered zeolites obtaining membranes. In fact, it is possible to promote zeolite crystallization inside a geopolymeric matrix by tuning pH, temperature and time of the geopolymerization reaction^23,24^. Geopolymers are inorganic silico-alluminate polymers, regarded as emerging sustainable ceramic materials, which currently attract great interest in the production of foams and membranes for use in several industrial applications^25–28^ due to their interesting combination of good mechanical properties, high chemical stability, large internal surface and high early strength.

Recently, a one-step procedure was successfully carried out realizing geopolymerization and crystallization under mild operating conditions^29^. An open cellular porosity was also induced by in-situ inorganic foaming process. It has been demonstrated that this route leads to the crystallization of two distinct zeolites, Na-A [LTA] and Na-X [FAU], obtaining a hierarchical porous monolith containing macro-, meso- and micro-pores.

In the present work, the above technique was adopted to produce a monolithic softener and its performance in hardness removal from tap water was evaluated. The kinetic mechanism controlling the softening process was studied and modelled by pseudo-first- and pseudo-second-order kinetic equations. Finally, the regeneration efficiency and reusability of the softener were assessed.

## Materials and methods

### Synthesis of the softener

**Figure 1:**
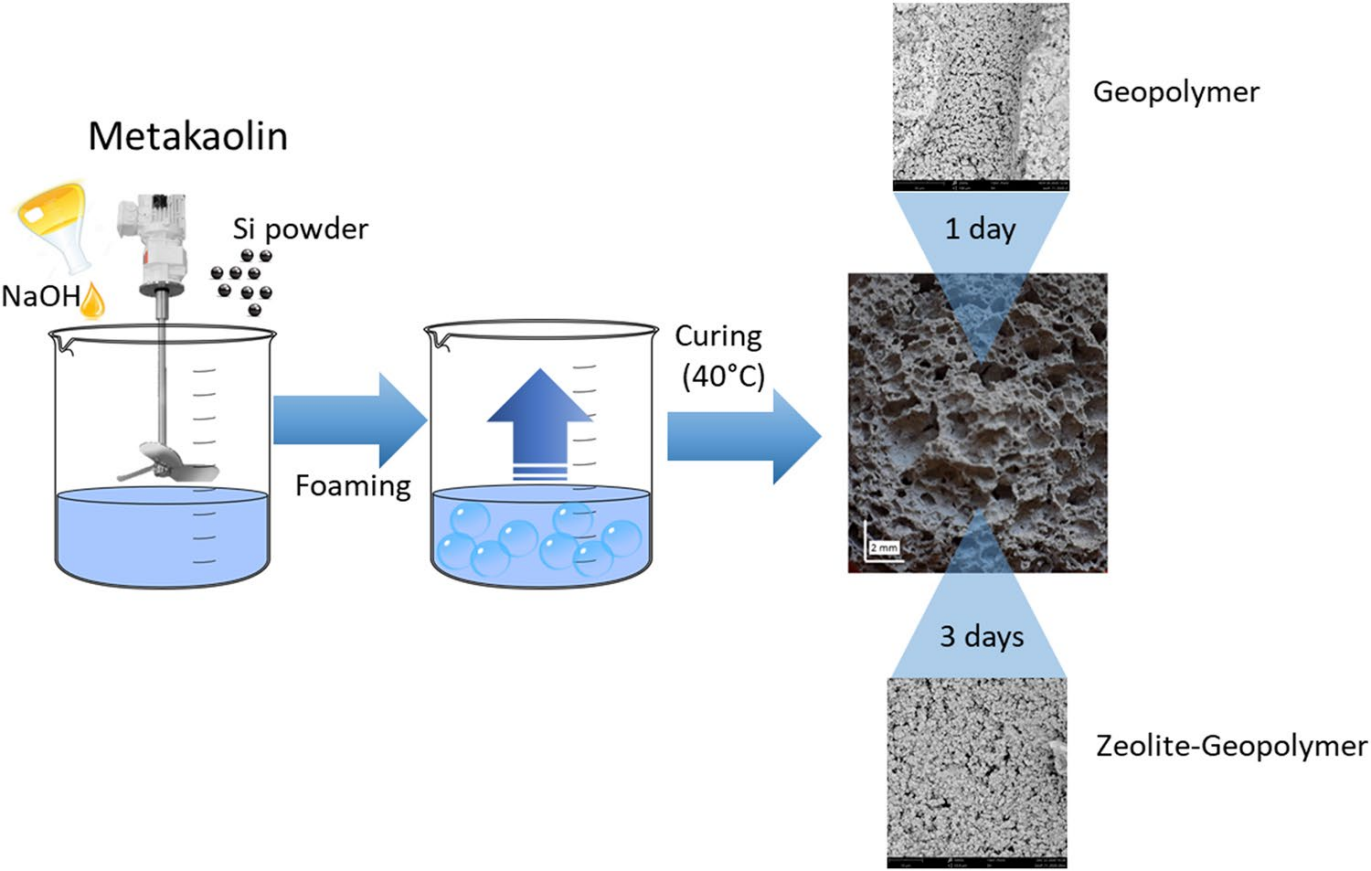
Softener preparation procedure.

The softener was obtained following the procedure reported in Liguori et al.^29^. Metakaolin, sodium hydroxide solution and silicon powder were chosen as raw materials. Metakaolin powder (MK, provided by Neuvendis) was used as silicon and aluminum source. The main features of the powder, provided by the producer, are the following: Al_2_O_3_ 41.90 wt%; SiO_2_ 52.90 wt%; K_2_O 0.77 wt%; Fe_2_O_3_ 1.60 wt%; TiO_2_ 1.80 wt%; MgO 0.19 wt%; CaO 0.17 wt%; specific surface area 12.69 m^2^ g^−1^; d_50_ = 3.64 μm. 10 M sodium hydroxide solution was used as alkaline activator (AA) and Silicon powder (1 wt% of the amount of metakaolin) was selected as pore-forming agent. A geopolymer gel precursor with SiO_2_:Al_2_O_3_ ratio = 2.14 and Na_2_O:SiO_2_ ratio = 1.0 was produced by intimately mixing MK and silicon and then adding a proper amount of AA. The gel was then put in a plastic mold at 40 °C and 100% relative humidity for a proper amount of time to promote the foaming and the subsequent geopolymer consolidation. Previous results proved that 1 day of curing was sufficient to obtain a pure geopolymeric sample, while a self-supporting zeolite was produced after longer curing times (starting from 3 days)^29^. Finally, the obtained sample was washed with deionized water up to pH < 10 to remove the residual sodium hydroxide and dried in an oven at 60 °C for 24 h. A scheme of the entire process is summarized in Fig. 1.

### Characterization of the softener

Zeolite content in the softener was checked by means of X-ray diffraction using a Panalytical X'Pert Pro diffractometer equipped with PixCel 1D detector (operative conditions: CuK_α1_/K_α2_ radiation, 40 kV, 40 mA, 2Θ range from 5 to 80°, step size 0.0131°2Θ, counting time 40 s per step). Sample morphology was assessed by scanning electron microscopy (SEM) using a Phenom Pro X Microscope on fracture surfaces. Density and porosity were calculated according to the European Standards UNI 11060:2003 and UNI EN 1936:2007.

Prismatic samples (16 × 4 × 4 cm) were also prepared for mechanical characterization. In particular, three-point flexural tests were carried out using a Tensometer 2020 device by Alpha Technologies, with a 5 kN load cell and a crossbar lowering speed of 1 mm min^−1^. On each of the two parts of samples obtained from the flexural tests a compression test was also performed using the same device with a 5 kN load cell and a crossbar lowering speed of 2 mm min^−1^.

The cation exchange capacity (CEC), i.e. the maximum amount of cations as milliequivalents exchanged per gram of substance, was evaluated by means of the Cross-Exchange method reported in de Gennaro et al.^30^. Accordingly, a complete Na → K exchange was performed by contacting a suitable amount of sample with 1 M KCl solution at a solid-to-liquid ratio = 2 g L^−1^ for 3 h at 40 °C under continuous stirring, then the liquid phase was separated by centrifugation, and sampled for further analysis. Such procedure was repeated 10 times. After that, the sample was washed, dried, and the total amount (meq g^−1^) of exchanged Na^+^cations was then calculated as the sum of the amounts exchanged after each cycle. A complete K → Na reverse exchange was then performed on the same sample and under the same operating conditions (using a 1 M NaCl solution), obtaining the total amount of exchanged K^+^ cations. The CEC was finally evaluated as the average of the two values. The cationic amount was evaluated by ICP optical emission spectroscopy (ICP-OES, Perkin-Elmer Optima 2100 DV).

### Evaluation of hardness removal

To study the water hardness removal in a real scenario, a weighed sample was put in contact with tap water at a solid-to-liquid ratio of 2.5 g L^−1^ under continuous stirring. The water used comes from the city of Naples and it is characterized by an average concentration of 126.9 ppm of calcium and 37.06 ppm of magnesium, corresponding to a hardness of 471.5 ppm of CaCO_3_ (47.15 °F). The hardness, in terms of calcium and magnesium concentration was evaluated before each adsorption run.

The kinetic of Ca^2+^ and Mg^2+^ removal has been evaluated. 10 mL withdrawals were taken at fixed times from 0 to 1440 min, the liquid was separated and the cation concentrations were determined by ICP. The pH was monitored during each run.

The percentage of Ca^2+^ (or Mg^2+^) removed was obtained as:

1 $$\frac{{C}_{0}- C\left(t\right)}{{C}_{0}}\times 100$$

where C(t) and C_0_ [mg L^−1^] are the cation concentration at time t and time t = 0, respectively.

To estimate the removal efficiency RE of calcium and magnesium, the maximum adsorption was calculated according to the following equation^31^:

2 $$RE=\frac{{C}_{i}- {C}_{f}}{m} \cdot V$$

where *C*_*i*_ [mg L^−1^] is the initial concentration of cation in the tap water, *C*_*f*_ [mg L^−1^] the concentration of cation at the end of the kinetic test, *m* [g] the sample mass and *V* [L] the volume of solution.

To evaluate the kinetic mechanism controlling the softening process the pesudo-first- and pseudo-second-order kinetic models were used. These kinetic models assumes that the limiting step of the process is the ion exchange reaction, that has a first-order or second-order kinetic, respectively. Accordingly, if the reaction follows a first order equation, the kinetic data can be described by the following equation:

3 $${q}_{t}={q}_{e}\left(1-{e}^{-{k}_{1}t}\right)$$

where *q*_e_ is the amount of cation removed at equilibrium (mg L^−1^) and k_1_ (min^−1^) is the pseudo-first-order rate constant. On the contrary, if the reaction follows a second order equation, the kinetic data can be described by the following equation:

4 $${q}_{t}=\frac{k{q}_{e}^{2}t}{1+k{q}_{e}t}$$

where *q*_e_ is the amount of cation removed at equilibrium (mg L^−1^) and k (mg L^−1^ min^−1^) is the pseudo-second-order rate constant.

Looking at an industrial scale application, the regeneration of saturated softener was studied. The regeneration of the softener was performed using NaCl solution at a solid-to-liquid ratio of 2.5 g L^−1^ under continuous stirring for 24 h. Two different concentrations of the regeneration solution (1 M and 3 M) were tested and also the effect of temperature was monitored at 25 or 60 °C. Monitoring the Ca^2+^ concentration released in the solution at fixed times, the regeneration precentage was calculated.

Finally, the reusability of the softener was assessed monitoring its removal ability after subsequent softening cycles (S/L = 2.5 g L^−1^ under continuous stirring for 4 h).

## Results and discussion

### Characterization of the softener

**Figure 2:**
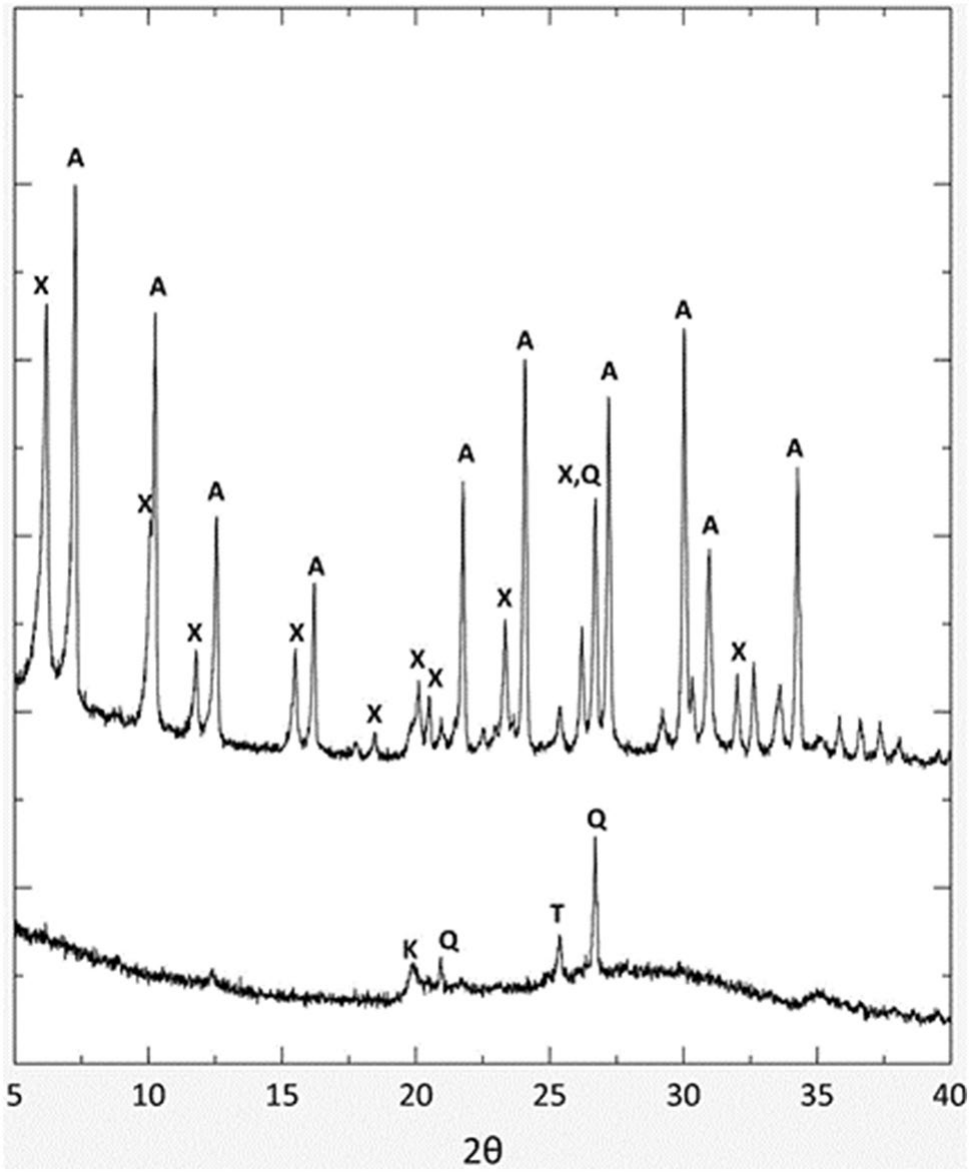
XRD pattern for one day (bottom) and three days (top) of curing. *A* zeolite LTA, *X* zeolite X, *Q* quartz, *K* feldspar, *T* titanium oxide.

A self-supporting zeolite (ZEOP) was obtained starting from 3 days of curing at 40 °C. Spectra reported in Fig. 2 showed the presence of two distinct crystal phases, identified as zeolites Na-A ([LTA], ICCD ref. code n. 00-039-0222) and 13X ([FAU], ICCD ref. code n. 01-083-2319), while in the one-day sample no crystalline phases were detected.

**Figure 3:**
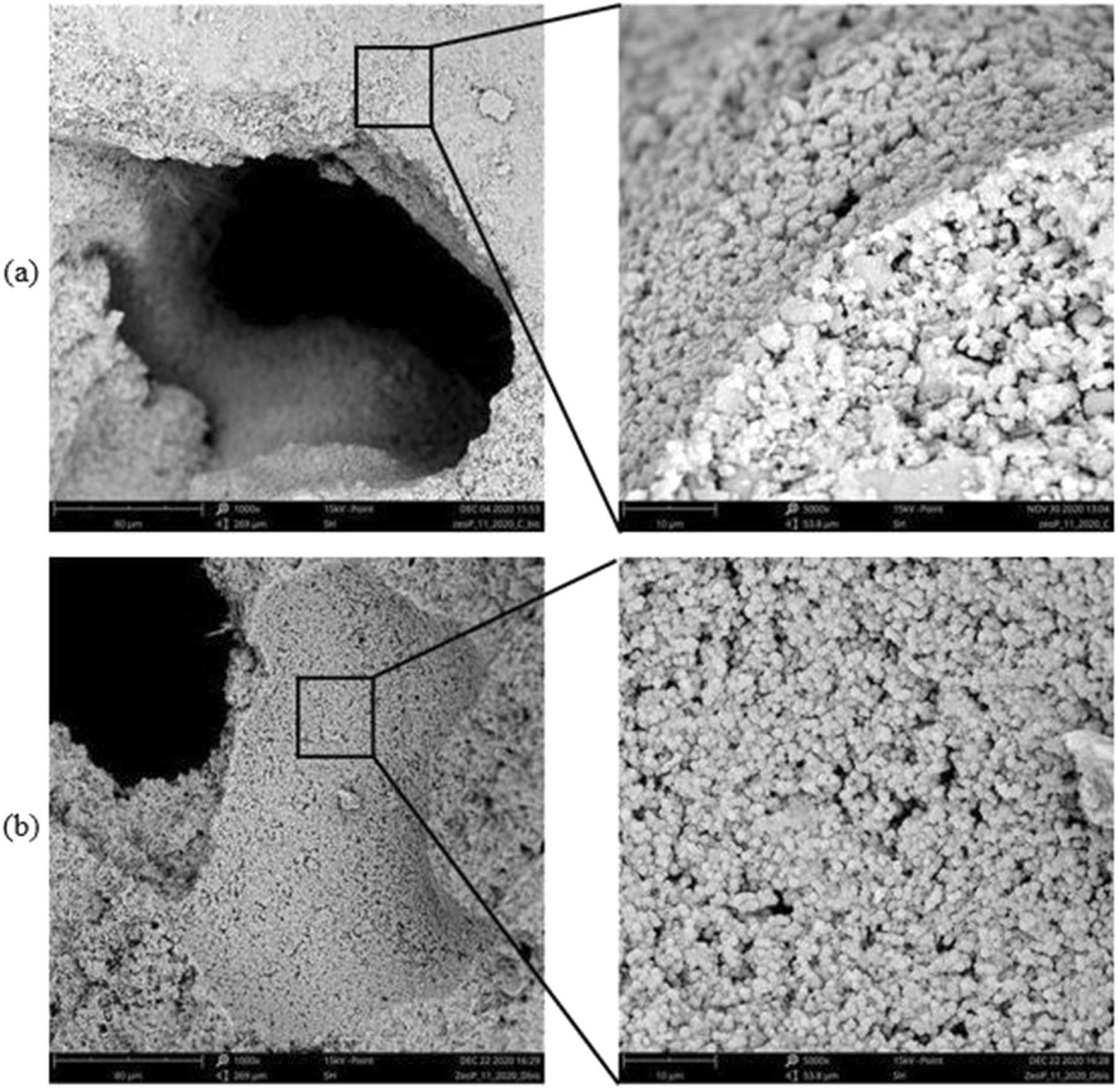
Microstructure of the sample after one day (**a**) and three days (**b**) of curing at different magnification (×1000 left and ×5000 right).

Figure 3 shows the evolution of the morphology of the softener at different magnification levels during the curing runs.

After 1 day the sample showed an amorphous structure typical of geopolymer (Fig. 3a). The presence of a relevant amount of nanosized zeolite crystals occurred from three days (Fig. 3b): the microstructure presented well-developed zeolite Na-A crystals with cubic like structure surrounded by smaller nanometric crystals with the typical morphology of FAU zeolites, as already revealed by XRD spectra.

As reported in Liguori et al.^29^ ZEOP showed a BET specific surface of about 189.6 m^2^ g^−1^ evaluated by N_2_ adsorption/desorption cycles at 77 K with a total specific pore volume of 475.05 mm^3^ g^−1^ at 400 MPa and a total specific pore area 11.10 m^2^ g^−1^, evaluated by Mercury Intrusion Porosimetry (MIP).

The macroporosity was evaluated by water absorption tests (Table 1). Confirming the role of the in-situ inorganic foaming, a similar open cellular porosity was induced (about 66%). Moreover, since the geopolymeric amorphous framework is responsible for the mechanical features of the softener, compressive and flexural strengths do not decrease after the crystallization of zeolites.

**Table 1:** Physical properties of the softener at different curing times.

Curing time	Open porosity [%]	Bulk density [g cm^-3^]	Compressive strength [MPa]	Flexural strength [MPa]
1 day	64.56 ± 1.96	0.57 ± 0.02	0.39 ± 0.072	0.33 ± 0.068
3 days	68.49 ± 2.54	0.53 ± 0.03	0.39 ± 0.071	0.31 ± 0.015

As expected, the geopolymeric sample showed a CEC value of 3.22 meq g^−1^, likely due to the presence of sodium cations, extra reticular and weakly bonded to the framework^32–34^. Nevertheless, after three days of curing a significant increase in CEC occurred (4.43 meq g^−1^), confirming the presence of zeolites, which possess a higher CEC value (the theoretical cation exchange capacities are 4.83 meq g^−1^ and 5.4 meq g^−1^ for X and A respectively).

### Water softening process

According to the previous characterization, the sample cured for three days (labeled as ZEOP) was selected for the hardness removal process.

**Figure 4:**
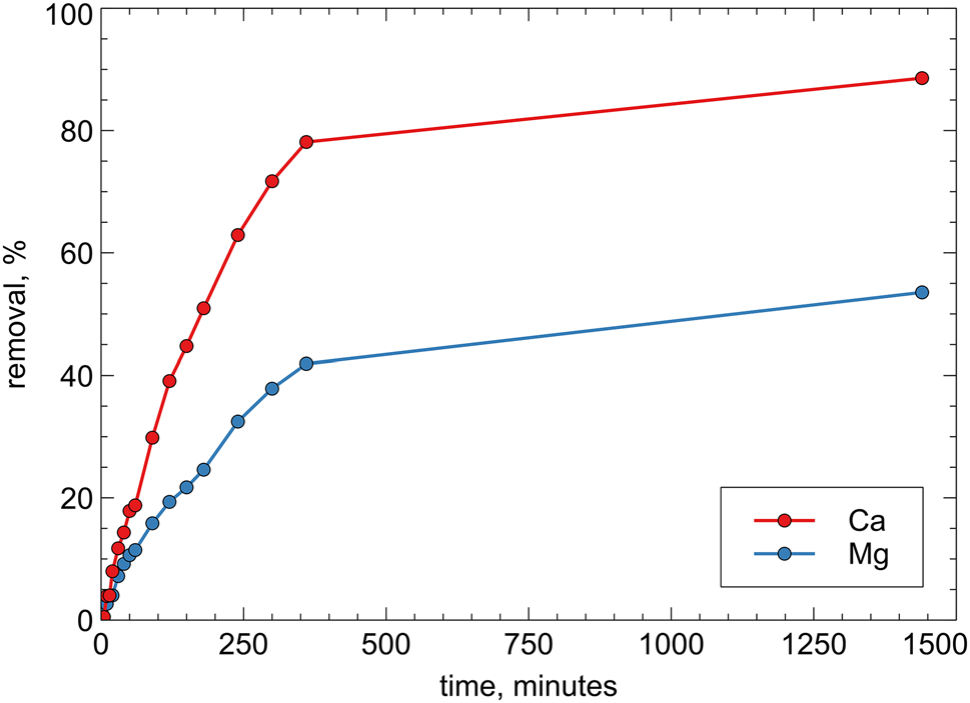
ZEOP calcium and magnesium uptake kinetic under uncontrolled pH conditions.

The uptake of calcium and magnesium, calculated by Eq. (1), is reported in Fig. 4.

The sample showed a good softening performance: after 240 min the uptake of calcium is about 60%, while after one day the removal reaches about 90%. For magnesium removal, after 240 min the uptake of magnesium is about 33%, while after one day about 54%.

These values show an uptake rate slower than that showed by pure powdery zeolites^18,19^, which is certainly due to the slower diffusion of the cations in the monolitic sample. Moreover, the pH variation of the solution during the softening process (ranging from 7 to 9) suggests that the process is due to a combination of cation exchange and precipitation phenomena.

**Figure 5:**
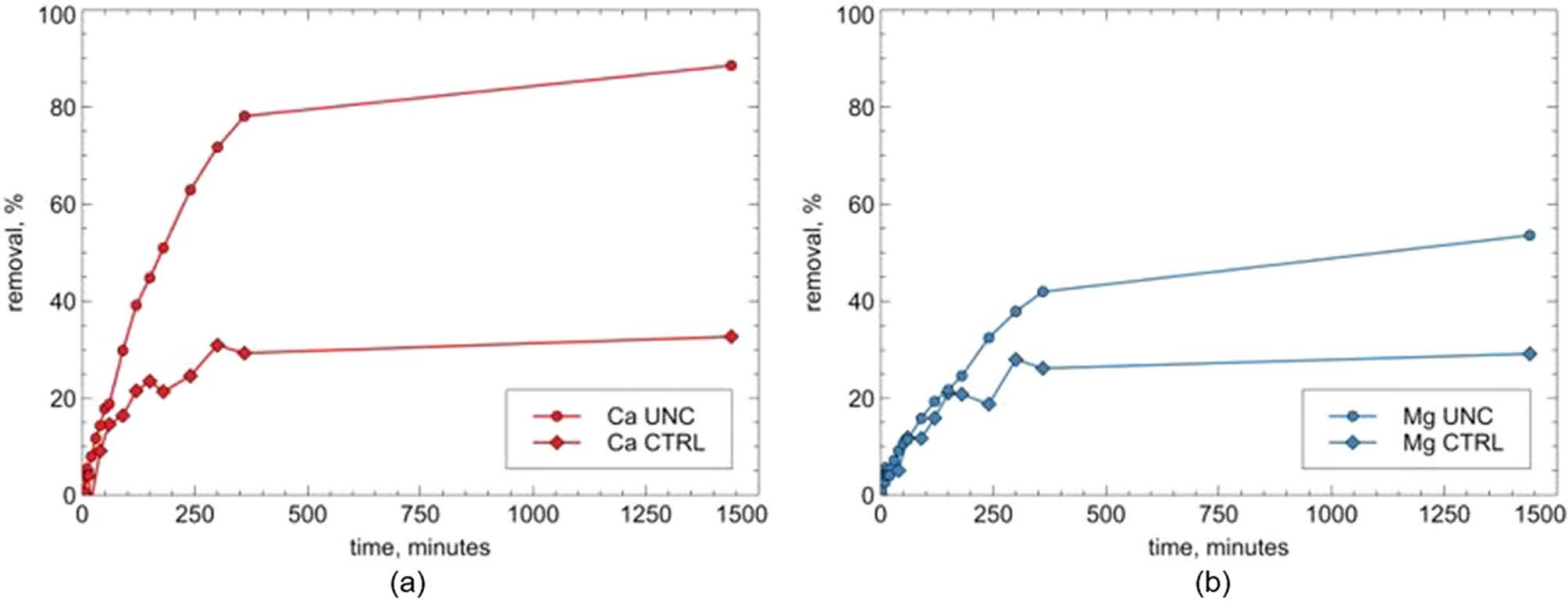
Calcium (**a**) and magnesium (**b**) kinetic curves for ZEOP samples under uncontrolled (UNC) and under controlled pH conditions, pH = 5, (CTRL).

In order to discriminate between these two phenomena, other softening runs were performed by keeping pH at 5 by means of dropwise nitric acid addition (Fig. 5).

Comparing the runs under different pH conditions (Fig. 5) suggests the use of the softener in dual mode, since when the ion-exchange is the only active process, lower efficiencies are attained. The removal efficiency RE, evaluated following Eq. (2), for the dual-mode softener reachs 49.02 mg g^−1^ for calcium and 7.94 mg g^−1^ for magnesium. To further prove the presence of a precipitation phenomenon, the softened water was filtered after both the removal runs. No residue was detected upon filtering the water softened under controlled pH condition. On the contrary, the water softened under uncontrolled pH condition left a white powder, which was subjected to XRD analysis. The results (see Figure S1 in Supporting Information) confirmed the presence of calcium carbonate (calcite, ICDD PDF-2 database record n. 01-083-0578), and thus proved the precipitation process.

**Figure 6:**
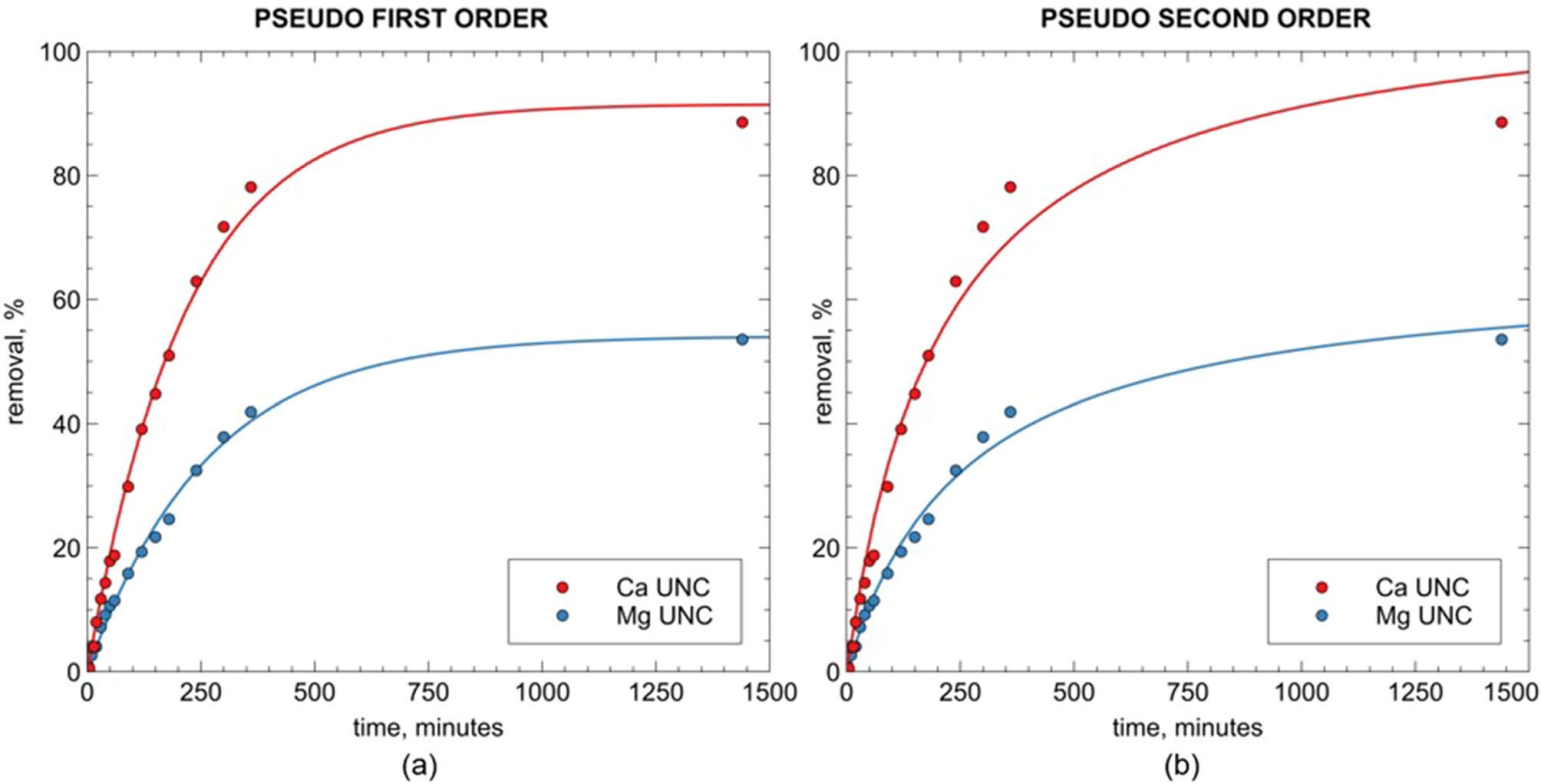
Modeling results for calcium and magnesium kinetic curves for ZEOP sample under uncontrolled pH conditions.

This is further confirmed by the modeling results. Overall, the kinetic curves were satisfactorily modelled either with the pseudo-first or the pseudo-second order kinetic equation (see the coefficient of determination values in Table 2). Nonetheless, the pseudo-first order kinetic model gave better results in interpreting the uncontrolled pH kinetic data (Fig. 6a).

**Table 2:** Kinetic pseudo-first-order and pseudo-second-order parameters for Ca^2+^ and Mg^2+^ removal on ZEOP samples.

Cation	Model	Parameter	ZEOP (uncontrolled pH)	ZEOP (pH = 5)
Ca^2+^	Pseudo-first order	q_e_ (mg g^-1^)	46.4 (91.5%)	19.2 (31.9%)
		k_1_ (min^-1^)	4.7 E^-03^	8.0 E^-03^
		R^2^	0.995	0.939
	Pseudo-second order	q_e_ (mg g^-1^)	55.9 (110.2%)	22.5 (37.4%)
		k_2_ (mg L^-1^ min^-1^)	4.3 E^-05^	2.5 E^-04^
		R^2^	0.978	0.930
Mg^2+^	Pseudo-first order	q_e_ (mg g^-1^)	8.0 (54.1%)	4.4 (28.9%)
		k_1_ (min^-1^)	3.8 E^-03^	6.9 E^-03^
		R^2^	0.994	0.930
	Pseudo-second order	q_e_ (mg g^-1^)	9.7 (65.4%)	5.1 (33.6%)
		k_2_ (mg L^-1^ min^-1^)	5.9 E^-05^	2.4 E^-04^
		R^2^	0.988	0.922

*Number in parentheses report the equilibrium uptake as the removed percentage of the initial cation concentration.

In fact, concerning the Ca^2+^ kinetic curve obtained under uncontrolled pH conditions, the pseudo-second order model seems to underestimate the uptake at relatively short times (250 to 500 min, see Fig. 6). On the contrary, the same model also overestimates the equilibrium uptake, as can be seen in Table 2, where the obtained Ca^2+^ q_e_ value for ZEOP sample was 110.2% (which is obviously not physically possible). This is likely due to the competing contributes of precipitation and ion exchange phenomena on the overall uptake process, which the equation does not take into account. Much better results were obtained with the pseudo-first order kinetic equation, which probably better interpret the former phenomenon in describing the softening process.

**Figure 7:**
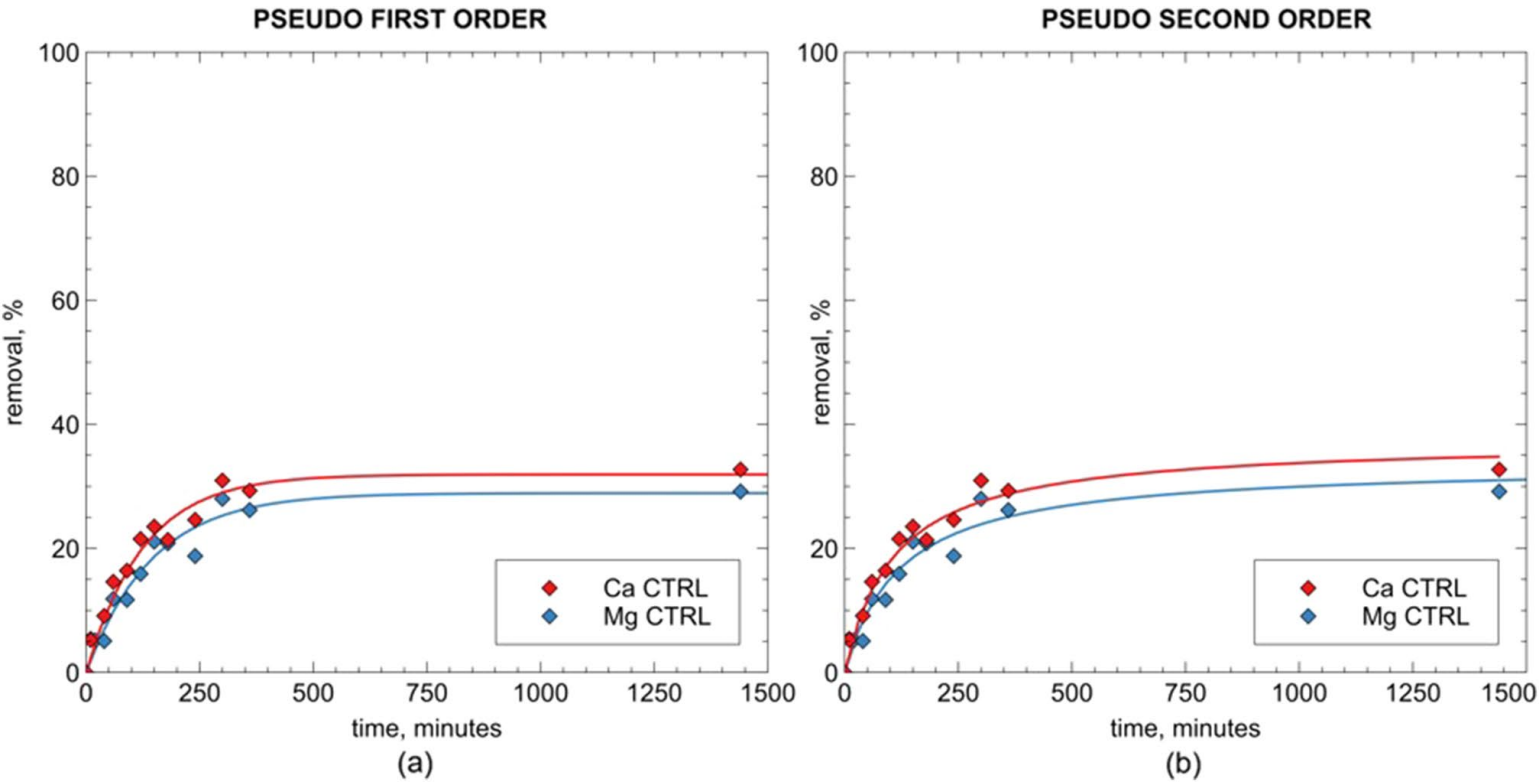
Modeling results for calcium and magnesium kinetic curves for ZEOP sample under controlled pH conditions (pH = 5).

The kinetic curves obtained at controlled pH (see Fig. 7), on the contrary, are well fitted also by the pseudo-second-order equation, once again proving that, in this case, the cation exchange is the only operating process.

Concerning the kinetic constants, the *k* constant for calcium uptake is higher than the magnesium one (Table 2). It means that samples remove the Ca^2+^ ions faster than the Mg^2+^ ions. Moreover, the *k* values obtained under uncontrolled pH are always lower than those obtained under controlled pH conditions: it shows that the ion exchange process is quite faster than the precipitation for both cations.

Removal efficiency attained were compared to the scientific literature (Table 3), which confirms that the proposed approach can compete with synthetic or natural materials.

**Table 3:** Removal efficiency of calcium and magnesium compared to the literature.

Sample	RE, mg g^-1^		References
	Ca^2+^	Mg^2+^	
ZEOP (uncontrolled pH)	49.02	7.94	This study
ZEOP* (pH = 5)	20.16	4.44	This study
Commercial Zeolite Na-A	17	–	^19^
Synthesized Zeolite Na-A	31	–	
Clinoptilolite	11	–	^35^
Sand Materials (Natural Zeolites)	41.2	–	^36^
Modified Bentonite coatings	14.63	14.63	^37^
Natural Pumice Stones Modified Pumice Stones	57.2 62.3	44.5 56.1	^13^

*At pH = 5 the softening is merely based on cation exchange.

### Regeneration and reuse of the softener

The regeneration ability of zeolite can improve the lifespan of the softener and consequently reduce the cost of the entire water treatment.

**Figure 8:**
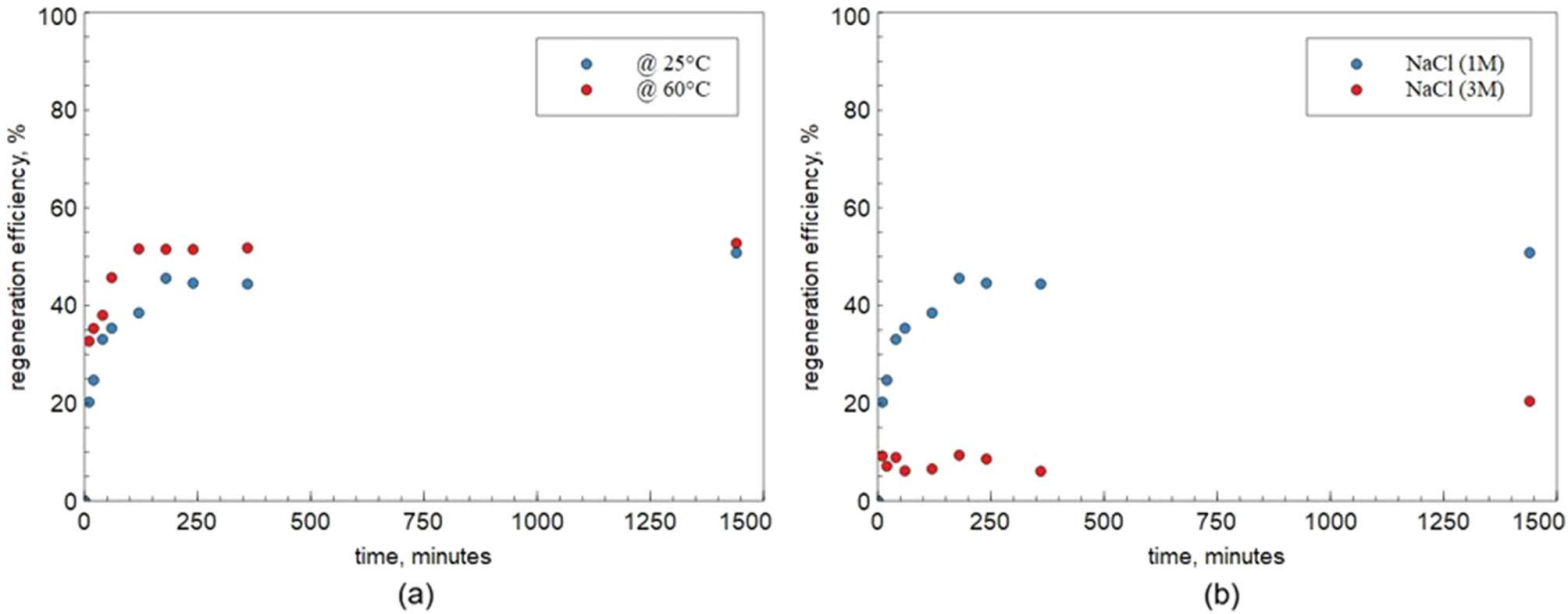
Regeneration efficiency of ZEOP (uncontrolled pH): (**a**) at different temperature and (**b**) at different NaCl concentration.

The results of regeneration efficiency using two different concentration of NaCl solution (1 M and 3 M) and two different temperatures (25 and 60 °C) are presented in Fig. 8.

Results indicate that using 1 M NaCl solution provided the best results and about 50% of the adsorbed Ca^2+^ was extracted, regardless of the temperature, after 4 h. On the contrary, the use of a more concentrated regeneration solution seems to hinder the calcium release from the softener lowering the regeneration efficiency (about 20%). Similar results were obtained for Mg^2+^.

**Figure 9:**
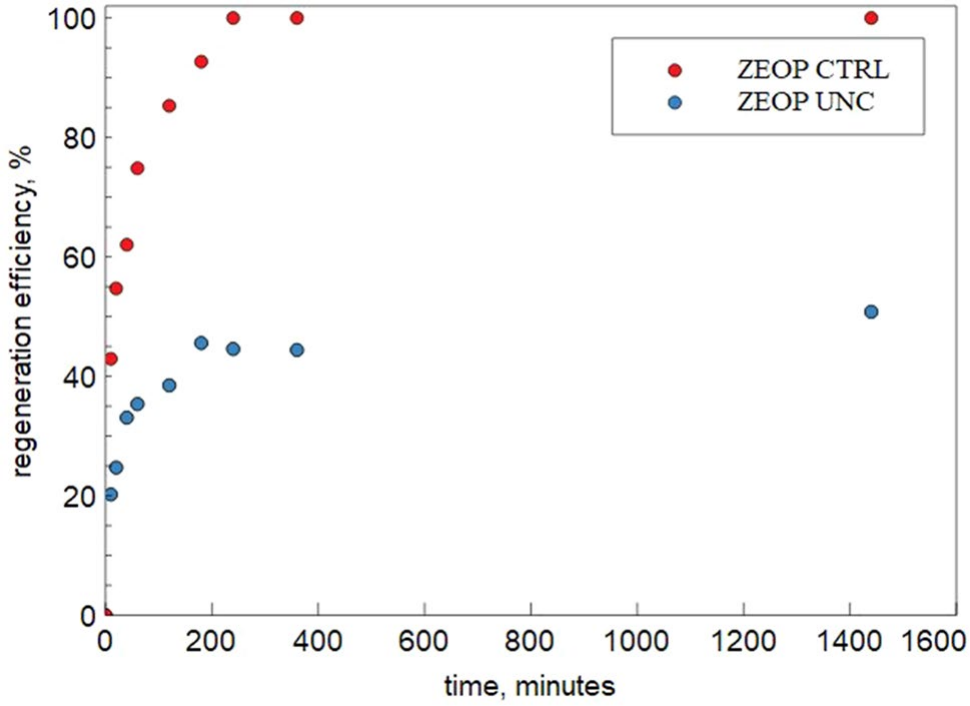
Regeneration efficiency of ZEOP UNC (uncontrolled pH) and ZEOP CTRL (pH = 5) at 25 °C with a 1 M NaCl solution.

When the ion-exchange is the only active process, for the ZEOP (pH = 5), the regeneration runs confirmed the complete reversibility of the softening process in 24 h with 1 M NaCl solution (Fig. 9).

**Figure 10:**
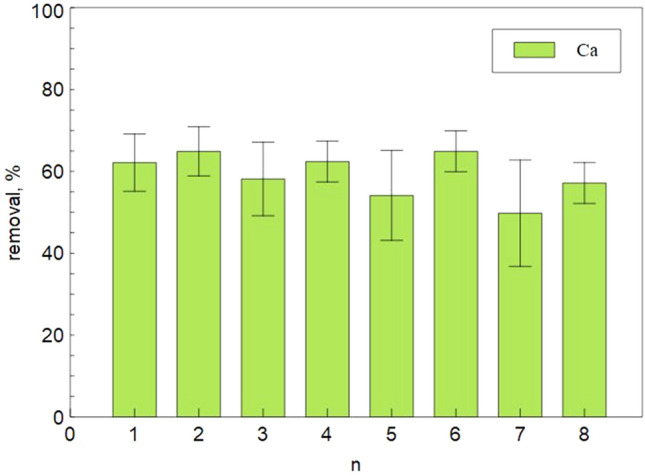
Removal efficiency after subsequent cycles of 4 h.

Another interesting opportunity is the reuse without washing or regeneration. it has been proved that the sample preserves its efficiency after 8 subsequent cycles (Fig. 10).

## Conclusions

Collected data show that it is possible to apply geopolymer – zeolite composites, obtained by Geopolymer Gel Conversion, as bulk type adsorbents in softnening processes.

Softening runs at different pH conditions demonstrate that the removal of calcium and magnesium is due to a combination of cation exchange and precipitation phenomena.

With respect to traditional carbonate hardness removal methods, such as lime-soda, a reduction in the volume of softening sludge can be achieved. At the same time, the presence of zeolites (LTA and FAU-X) makes possible a partial regeneration of the softener and give it an additional skill of water remediation thanks to the well-known selectivity of zeolites towards heavy metals. These promising results, combined with the reusability of the softeners, suggest the real possibility of using an alternative method in water softening process.

## Supplementary Information

Supplementary Information.
