# Supplementary material for: Zeolite-based monoliths for water softening by ion exchange/precipitation process
Source: Sci Rep. 2022 Mar 7;12:3686. doi: 10.1038/s41598-022-07679-2 (PMC8901846; doi:10.1038/s41598-022-07679-2)
Supplement: Supplementary file 1 — Supplementary Information. [file 41598_2022_7679_MOESM1_ESM.docx]

**A DUAL MODE SOFTENER MADE BY SELF-SUPPORTING ZEOLITES**

A. Campanile^1^, B. Liguori^1*^, C. Ferone^2^, D. Caputo^1^, P. Aprea^1^

^1^ Applied Chemistry Labs-Department of Chemical, Materials and Industrial Engineering, University of Naples Federico II, Naples, Italy

^2^ Materials Science and Engineering Research Group MASERG, Department of Engineering, University of Naples Parthenope, Naples, Italy


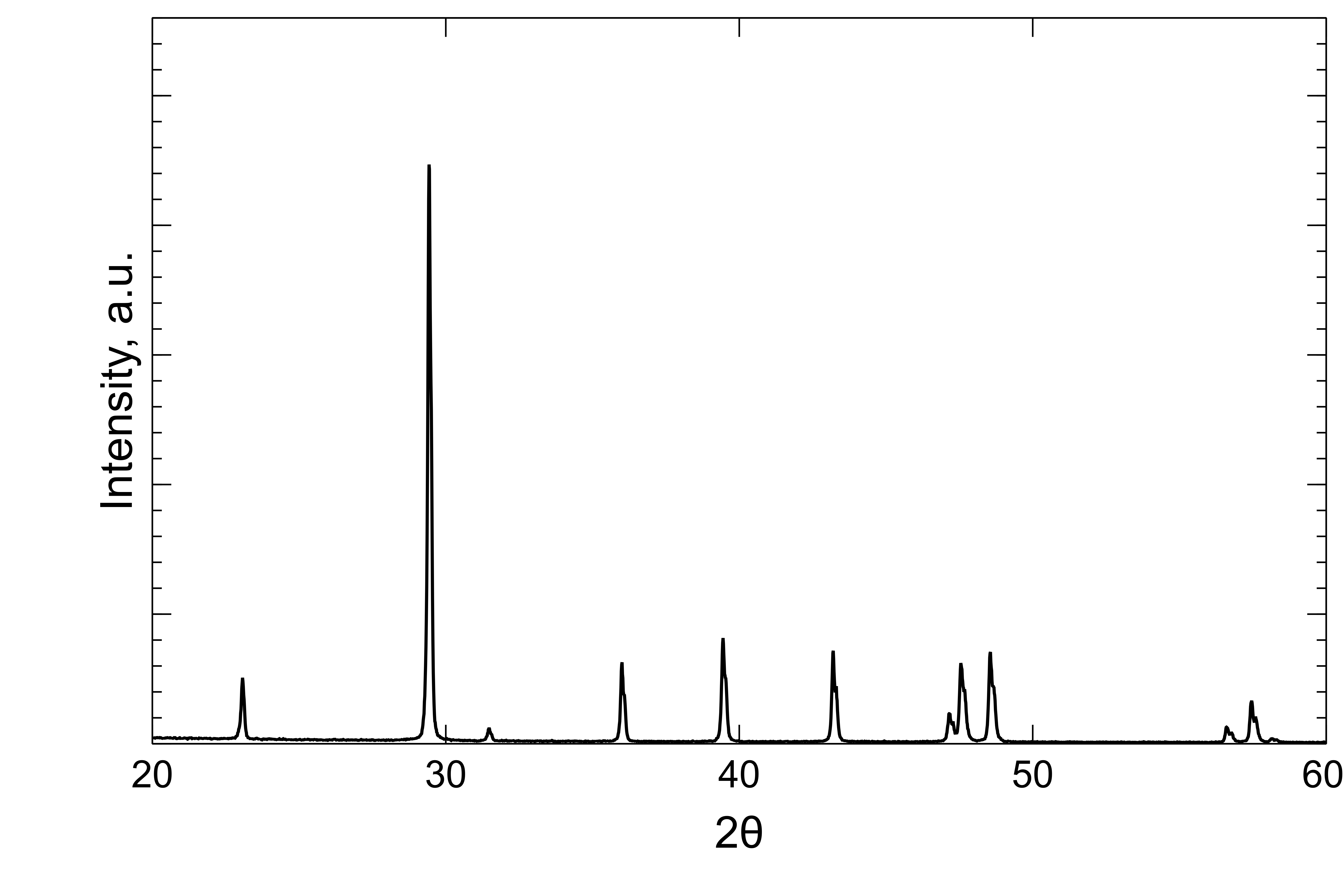


Figure S1 – XRD diffraction pattern of precipitate recovered after softening under uncontrolled pH conditions.
